# Predictive factors of radiation-induced skin toxicity in breast cancer patients

**DOI:** 10.1186/1471-2407-10-508

**Published:** 2010-09-23

**Authors:** Miao-Fen Chen, Wen-Cheng Chen, Chia-Hsuan Lai, Chao-hsiung Hung, Kuo-Chi Liu, Yin-Hsuan Cheng

**Affiliations:** 1 Department of Radiation Oncology, Chang Gung Memorial Hospital, Chia-Yi, Taiwan; 2 Chang Gung University College of medicine and Chang Gung Institute of Technology, Tao-Yuan, Taiwan

## Abstract

**Background:**

To assess the factors affecting the incidence of radiation-induced dermatitis in breast cancer patients treated with adjuvant 3 D conformal radiotherapy by the analysis of dosimetry and topical treatments.

**Methods:**

Between September 2002 and July 2009, 158 breast cancer patients were treated with adjuvant 3 D conformal radiotherapy after undergoing surgery. Before November 2006, 90 patients were subjected to therapeutic skin care group and topical corticosteroid therapy was used for acute radiation dermatitis. Thereafter, 68 patients received prophylactic topical therapy from the beginning of radiotherapy. The two groups did not differ significantly in respect of clinical and treatment factors. Furthermore, the possible mechanisms responsible for the effects of topical treatment on radiation-induced dermatitis were investigated *in vivo*.

**Results:**

The incidence of radiation-induced moist desquamation was 23% across 158 patients. Higher volume receiving 107% of prescribed dose within PTV (PTV-V_107%_; >28.6%) and volume receiving 110% of prescribed dose within treated volume (TV-V_110%_; > 5.13%), and no prophylactic topical therapy for irradiated skin, were associated with higher incidence of acute radiation dermatitis. The protective effect of prophylactic topical treatment was more pronounced in patients with TV-V_110% _> 5.13%. Furthermore, using irradiated mice, we demonstrated that topical steroid cream significantly attenuated irradiation-induced inflammation, causing a decrease in expression of inflammatory cytokines and TGF-beta 1.

**Conclusion:**

TV-V_110% _> 5.13% may be an important predictor for radiation induced dermatitis. Prophylactic topical treatment for irradiated skin can significantly improve the tolerance of skin to adjuvant radiotherapy, especially for patients with higher TV-V_110%_.

## Background

Radiotherapy (RT) is commonly used as an adjuvant modality in the treatment of breast cancer [1,2]. Adjuvant chest wall irradiation for high risk breast cancer patients receiving modified radical mastectomy (MRM), and whole breast irradiation for patients after a breast-conserving surgery (BCS), are known to decrease loco-regional recurrence and improve overall survival. However, acute and chronic toxicities have been noted in patients treated with adjuvant breast or chest wall RT, including skin (30~40%), lung (3/%) and heart toxicity (1.5%) [3-5]. Traditionally, adjuvant RT for breast cancer patients after surgery is delivered using conventional tangential fields. An important concern with conventional RT is dose inhomogeneity resulting in irradiation of more normal tissue. In recent years promising RT techniques have been developed for various malignancies, including three-dimensional conformal radiotherapy (3D-CRT), IMRT and tomotherapy. These have improved dose homogeneity and conformity, and are associated with relatively low risks for toxicity compared with conventional RT technique [6-8].

RT-induced skin toxicity is a prominent clinical problem affecting the majority of breast cancer patients receiving adjuvant RT and can lead to temporary or permanent cessation of treatment. Severe skin reactions may be painful, lead to localized or occasionally systemic infection, and cause permanent scarring. The incidence of RT-related toxicity may be reduced by refinements in radiation techniques, such as improving dose conformity and dose homogeneity within the irradiated area. It is reported that breast IMRT could reduce approximately 15-20% moist desquamation of the irradiated skin by delivering a more homogenous dose of radiation through the breast and efficiently removing the radiation hot spots [9,10]. Accordingly, better understanding of dosimetric parameters, which may be related to acute skin toxicity in these women, will help to improve treatments in the future. Therefore, we present an analysis of the correlation between radiotherapy parameters and acute skin toxicity in breast cancer patients treated with 3D-CRT at our institution.

It is recommended that skin in the irradiated area be kept clean and free from trauma. Many physicians commence topical therapy at the clinical onset of radiation dermatitis but there is no consensus regarding the most appropriate timing or agents for topical therapy in such instances. Recently, some agents including creams containing urea and steroid, have been investigated and showed significant effects to reduce radiation induced dermatitis [11-13]. However, the treatment of acute radiation dermatitis still varies between different oncology center. Therefore, we evaluated whether prophylactic topical therapies can decrease the incidence of radiation-induced skin toxicity in a clinical setting as well as *in vivo*.

## Methods

### Characteristics of patients and treatment

This retrospective study was approved by the Institutional Review Board, and a waiver of informed consent was obtained. The patient data consisted of women who had undergone surgery for breast cancer followed by adjuvant 3D-conformal radiotherapy in our department between September 2002 and July 2009. Patients treated before November 2006 did not receive topical therapy for the skin until the onset of radiation dermatitis (therapeutic skin care group). Patients treated after this date underwent prophylactic topical therapy, including steroid cream (0.1% mometasone) and barrier film spray (3M™Cavilon No-sting Barrier Film) (prophylactic skin care group). Prophylactic medication was applied to the treatment field every three days from the start of RT [12]. In general, adjuvant 3D-CRT was prescribed for 50.4 Gy of external beam radiotherapy in 28 fractions. If required, adjuvant chemotherapy was performed sequentially rather than concurrently with radiotherapy in these patients (105 patients). Among these irradiated patients, there were 92 patients received hormone therapy. The Radiation Therapy Oncology Group (RTOG) scale was used to evaluate acute skin toxicity during radiation treatment at weekly clinical examinations, which continued for three weeks after the end of radiotherapy. Grade 2 skin toxicity is described in the RTOG Acute Morbidity Scale as, "tender or bright erythema, patchy moist desquamation/moderate edema, and Grade 3 as, "confluent moist desquamation other than skin folds, pitting edema". The correlation between acute radiation dermatitis and examined risk factors was calculated as the percentage of patients with moist desquamation (including grade 2 and grade 3 RTOG acute skin toxicity).

### Radiotherapy planning

**Figure 1:**
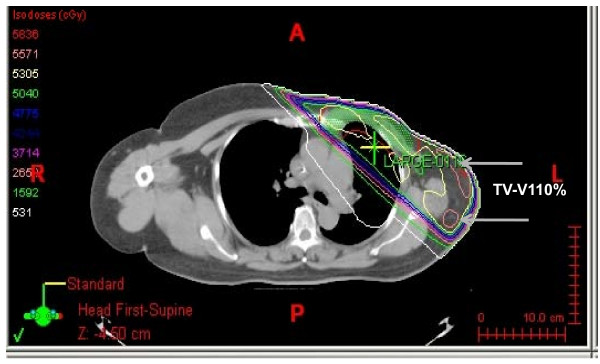
**3D-conformal radiation therapy isodose distributions are presented for a representative patient**.

Radiotherapy was planned using the Eclipse Planning System (version 7.1.35, Varian Medical System, Palo Alto, CA), and treatment was delivered using Varian 21EX. All patients were treated with 3 D conformal radiotherapy. For radiation therapy with 3D-CRT, a customized immobilization device was developed which encompassed the upper extremities, head, neck and chest, to minimize variability in the daily setup. The clinical target volume (CTV) was contoured on the individual axial CT slices with 5 mm slice thickness of each patient. The CTV was expanded by 10 mm, but within 3 mm of the skin surface, to create the planned target volume (PTV). Treatment plans were developed by applying tangential photon fields set up isocentrically, with or without individually weighted segmental fields superimposed on the tangential fields and 1-2 coplanar, different gantry angle fields. Wedges were used in almost all cases. Our planning goals were to provide a homogenous PTV dose of 50.4 Gy, while minimizing the dose delivered to the lung, heart and contralateral breast. Furthermore, to evaluate the effects of dose inhomogeneity on acute skin toxicity, we analyzed several dosimetric factors including the planning target volume (PTV), PTV-V_107% _(percent volume receiving 107% of prescribed dose within PTV) and TV-V_110% _(percent volume receiving 110% of prescribed dose within treated volume (TV)) to identify the hot spot area within and outside the target. The definition of treated volume is that volume enclosed within the prescribed dose, and the areas receiving excessive dose, especially >10% of prescribed dose, are known as radiation hot spot. Figure 1 shows the isodose distribution of a representative patient, illustrating the area of PTV-V_107% _and TV-V_110% _related to the location of moist desquamation.

### Statistical methods

The χ^2 ^test was utilized to compare acute skin toxicity between different sample groups, and to analyze associations between acute toxicity, dosimetric parameters and clinical characteristics. Statistical significance was assumed at p < 0.05.

### Mice, radiation and topical therapy

Thirty male BALB/c mice, aged between 8 and 10 weeks old, were purchased from the National Science Council, Taiwan. Protocols relating to animal experimentation were approved by the Chang Gung Memorial Hospital Laboratory Animal Center. For irradiation experiments, anesthetized mice restrained in modified Perspex tubes and covered with a 0.3 cm bolus, received a irradiation dose of 15 Gy in a single fraction to the skin by 6 MeV electron from a linear accelerator [14]. Unirradiated mice were subjected to the same conditions but were not exposed to the radiation source (sham-irradiation). For topical treatment the mice were divided into three groups: C-mice, which received no topical treatment; S-mice, treated with topical steroid cream (0.1% mometasone) (cream was applied on the irradiated skin immediately after initial treatment and once every two days after irradiation); and F-mice, which received topical treatment by Barrier Film Spray (topical film spray was applied on the irradiated skin immediately after initial treatment and once every two days after irradiation).

### RNA isolation and real-time RT-PCR

At the indicated times after irradiation, three mice from each group were sacrificed by cervical dislocation, and irradiated skin was dissected and stored at -80°C pending analysis [15]. Specific Assay-on-Demand Gene Expression Assay mixes (including primer and Taqman MGB probes) for IL-1α/β, IL-6, TNF-α and TGF-β1 were used for real-time PCR (Applied Biosystems, Foster, CA, USA). The mRNA (2 μg) was reverse-transcribed with random primer to obtain the first cDNA strand. The first strand cDNA was amplified through 40 cycles (95°C for 15 s and 60°C for 1 min) with the TaqMan Universal PCR Master mix and the specific Assay-on-Demand Gene Expression Assay mix for each gene according to the manufacturer's instructions.

### Immunoblotting

Equal amounts of protein were loaded on to SDS-PAGE gels. After electrophoresis, the proteins were transferred to nitrocellulose membranes. The membranes were incubated with antibodies specific for TGF-β1, IL-6, MCP-1 and COX-2 (Santa Cruz Biotechnology, Inc), followed by incubation with horseradish peroxidase-conjugated secondary antibodies. Signals were detected using enhanced chemiluminescence. To normalize protein loading, the membrane was re-probed with mouse anti-r-tubulin antibody (1:1000).

### Immunochemical staining

Cellular aspects of inflammation were measured in skin tissue samples using immunochemical staining. Experimental and control mice were sacrificed by cervical dislocation 20 days after exposure to 15 Gy irradiation. The tissues were fixed in 10% buffered formalin, paraffin-embedded and sectioned at an average thickness of 5 μm. Briefly, samples were incubated overnight with goat anti-mouse TGF-β1 antibody (Santa Cruz Biotechnology, Inc., Santa Cruz, CA, USA) diluted 1:20 in 0.01 M RPMI at room temperature. After washing three times with PBS, the sections were incubated with biotinylated anti-goat IgG (1:100) for 10 min followed by peroxidase-avidin staining. Samples were washed with PBS, followed by addition of 3-amino-9 ethylcarbazole.

## Results

### Patients and treatment

One hundred and fifty-eight patients met the study criteria and completed the planned course of treatment. Ninety patients underwent chest wall irradiation and 68 patients received whole breast irradiation. The mean age (±SD) of the overall study population was 50 ± 11 (range 24-86) years. The incidence of moist desquamation was 23% of the total study population, 26% for patients who underwent chest wall irradiation and 19% for those that received whole breast irradiation. Among 37 patients developed moist desquamation,34 patients appeared grade 2 and 3 had grade 3 radiation dermatitis. The details of the treatment parameters are listed in Table 1. The median PTV-V_107% _and TV-V_110% _within the 158 patients were 28.6% and 5.13% respectively. In comparison of dosimetric parameters between the 2 groups, the mean dose, target coverage and dose inhomogeneity did not differ significantly between patients receiving chest wall irradiation or whole breast irradiation (Table 1). Furthermore, we divided the 158 patients into the therapeutic skin care group (comprising 80 patients) and the prophylactic skin care group (comprising 78 patients, including 35 treated with steroid ointment and 43 treated with barrier film spray). As shown in Table 2, dose inhomogeneity (indicated by PTV-V_107% _and TV-V_110%_) and surgery type did not differ significantly between the two groups, but the incidence of moist desquamation was significantly greater in the therapeutic skin care group than in the prophylactic skin care group (30% versus 16.6%, *p*=0.048). Furthermore, within the prophylactic skin care group there was no statistical difference in the incidence of radiation dermatitis between those patients treated with topical steroid cream and those treated with barrier film spray (14.2% versus 18.6%, *p*=0.61).

**Table 1:** Clinical and treatment characteristics of patients

	Total	MRM	BCS	*P *value
**Patients (No)**	158	90	68	
**Age**				
Mean±SD (y/o)	50 ± 11	56 ± 11	48 ± 10	
Range	24-86	26-86	24-78	
**Acute skin toxicity**				
Grade 2	34	23	11	0.27
Grade 3	3	1	2	
**CTV**				
Volume (c.c)	192 ± 59	169 ± 53	226 ± 63	
D _mean _(Gy)	53.2	53.1	53.3	
D _median _(Gy)	53.9	54.5	53.5	
V_100% _(%)	98	98	99	
**PTV**				
Volume (c.c)	413 ± 113	345 ± 102	504 ± 124	
D _mean _(Gy)	52.5	52.5	52.5	
D _median _(Gy)	53.1	53.0	53.0	
V_100% _(%)	94	93	95	
**PTV-V_107 _(**Median; cc)	101	81	132	
**PTV-V_107%_**,				
Median (%)	28.6	24.5	31.5	
>Median (28.6) (No)	79	40	39	
< = Median (No)	79	50	29	0.11
**TV-V_110 _(**Median; cc)	35	32	36	
**TV-V_110%_**				
Median (%)	5.13	5.59	4.37	
>Median (5.13) (No)	79	48	31	
< = Median (No)	79	42	37	0.33

*Abbreviation*: MRM= modified radical mastectomy, BCS= breast-conserving surgery D_mean _= mean received dose for target; D_median _= median received dose for target

V_100% _= percent volume receiving the prescribed dose

PTV-V_107% _= percent volume receiving 107% of prescribed dose within PTV

Treated volume (TV) = the tissue volume which received the prescribed dose

TV-V_110% _= percent volume receiving 110% of prescribed dose within TV

Data are presented as mean with standard deviation

**Table 2:** Treatment and dosimetry characteristics of patients between the two groups

	Therapeutic skin care	Prophylactic skin care	*P *value
**Patients (No)**	80	78	
**Surgery type**			
MRM	45	45	
BCS	35	33	0.85
**PTV volume**			
>Median (No)	36	43	
< = Median (No)	44	35	0.20
**Moist desquamation (grade 2+grade 3)**			
Yes	24	13	
No	56	65	0.048
**PTV-V_107%_**,			
>Median (No)	38	41	
< = Median (No)	42	37	0.52
**TV-V_110%_**			
>Median (No)	40	39	
< = Median (No)	40	39	1

*Abbreviation*:

PTV-V_107% _= percent volume receiving 107% of prescribed dose within PTV

Treated volume (TV) = the tissue volume which received the prescribed dose

TV-V_110% _= percent volume receiving 110% of prescribed dose within TV

### Acute toxicity

Univariate analysis of the data demonstrated that higher PTV-V_107% _(>28.6%), higher TV-V_110% _(> 5.13%) and no prophylactic topical therapy for irradiated skin, were significantly associated with moist desquamation (Table 3), and TV-V_110% _still possessed predictive power on the incidence of radiation dermatitis in multivariate analysis (Table 3B). Therefore, we used the median of TV-V_110% _as a cut-off value to divide the 158 patients into group I (comprising 79 patients with TV-V_110%_≤ 5.13%) and group II (comprising 79 patients with TV-V_110% _> 5.13%), and further analyzed the risk factors associated with higher grade skin toxicity for each group. We found no significant association between the incidence of acute skin toxicity and surgery type, PTV-V_107% _and prophylactic skin care or not for group I (Table 4A). In group II (patients with TV-V_110% _> 5.13%), prophylactic topical therapy significantly decreased the incidence of higher grade skin toxicity (*p*=0.008) (Table 4B).

**Table 3:** Univariate analysis to determine factors associated with higher grade radiation- induced dermatitis for 158 irradiated patients

Variables	P value		
Surgery type (MRM versus BCS)	0.266		
Hormone therapy (Yes versus no)	0.120		
PTV volume (< = median versus > median)	0.112		
PTV-V_107%_ (< = median versus > median)	0.039		
TV-V_110%_ (< = median versus > median)	0.000		
Skin care (prophylactic versus therapeutic)	0.048		

**Multivariate analysis to determine factors associated with higher grade radiation- induced dermatitis for 158 irradiated patients**			

**Variables**	**Odd ratios**	**95%** **confidence interval**	**p**

Surgery type	1.4832	0.6449-3.4107	0.3536
PTV volume	1.0637	0.4846-2.3347	0.8776
PTV-V_107%_	0.8488	0.4014-1.7951	0.6680
TV-V_110%_	0.1037	0.0305-0.3528	0.0003
Skin care	1.8679	0.9025-3.8662	0.0923

*Abbreviation*:

MRM= modified radical mastectomy

BCS= breast conservative surgery

**Table 4:** Univariate analysis to determine factors associated with higher grade radiation- induced dermatitis for 79 irradiated patients with TV-V_110% _< = Median

Variables	P value
Surgery type (MRM versus BCS)	0.577
PTV volume (< = median versus > median)	0.701
PTV-V_107%_ (< = median versus > median)	0.210
Skin care (prophylactic versus therapeutic)	0.547

**Univariate analysis to determine factors associated with higher grade radiation- induced dermatitis for 79 irradiated patients with TV-V_110% _>Median**	

**Variables**	**P value**

Surgery type (MRM versus BCS)	0.491
PTV volume (< = median versus >median)	0.183
PTV-V_107%_ (< = median versus >median)	0.700
Skin care (prophylactic versus therapeutic)	0.008

### Effects of topical treatment on the radiation-induced inflammatory mediator

**Figure 2:**
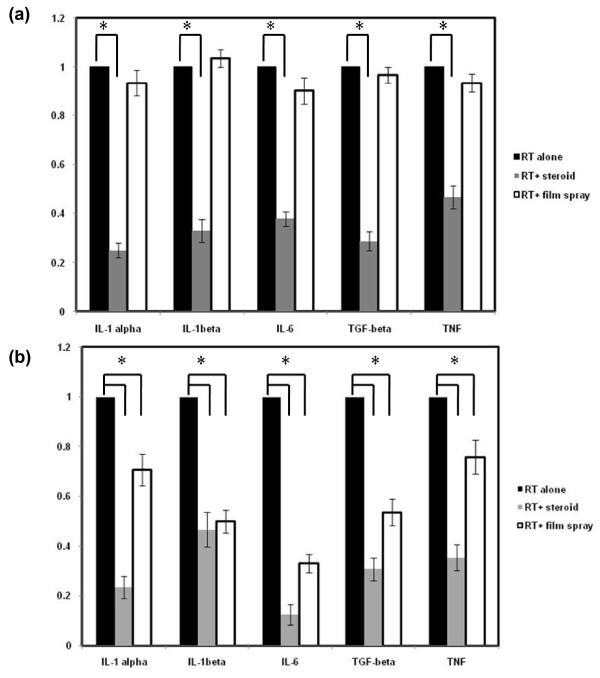
**Effect of topical treatments including steroid and barrier film spray on pro-inflammatory cytokines in skin and subcutaneous tissues after irradiation by real-time RT-PCR**. The mRNA levels of the cytokines TNF-α, IL-6, IL-1α/β and TGF-β were quantified by real-time RT-PCR. RNA were extracted from murine tissues (a) 24 h; (b) 7 days after irradiation. The results were normalized to the value of irradiated mice. The y-axis shows the RNA ratio of each target gene divided by that in the irradiated mice. Columns, means of 3 separate experiments; bars, SD. *, P < 0.05. Twenty- four hours after irradiation, topical steroid cream significantly attenuated the increase of pro-inflammatory cytokines in cutaneous tissues, but barrier film spray had no effect Furthermore, both topical treatments alleviated the RT-induced inflammatory response, and that steroid cream had a greater effect than barrier film spray 7 days after irradiation.

**Figure 3:**
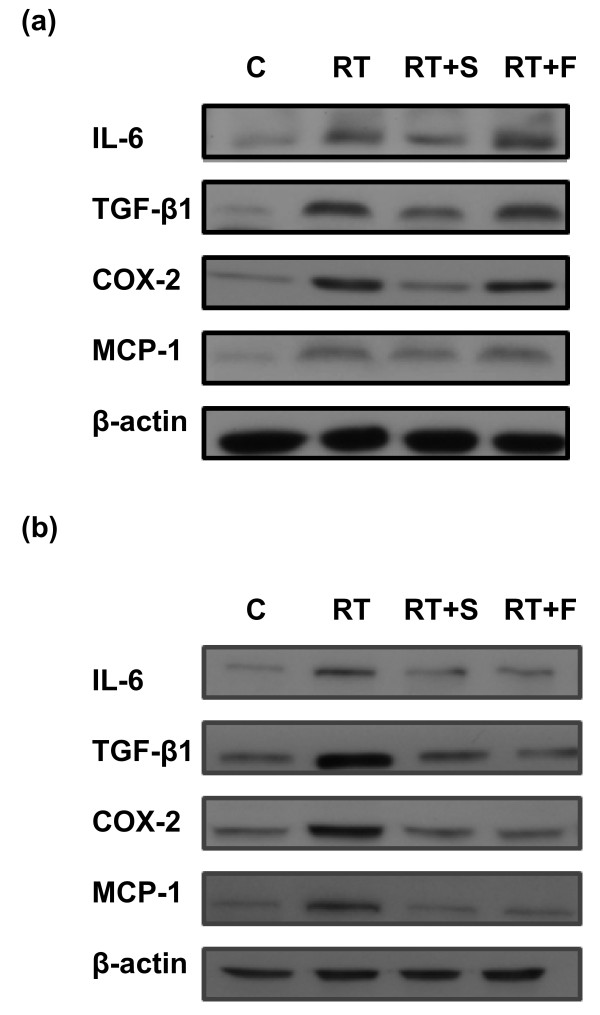
**Effect of topical treatments including steroid and barrier film spray on pro-inflammatory cytokines in skin and subcutaneous tissues after irradiation by Western blotting analysis**. Expressions of COX-2, IL-6, TGF-β1 and MCP-1protein in irradiated murine skin and subcutaneous tissue with or without topical treatment including steroid cream and barrier film spray. Proteins were extracted form murine tissues (a) 24 h; (b) 7 days after irradiation. (C, control; RT, irradiation; RT+S, steroid cream plus irradiation; RT+F, barrier film spray plus irradiation). Triplicate experiments were performed for the analysis. Twenty- four hours after irradiation, topical steroid cream significantly attenuated the increase of COX-2, IL-6, TGF-β1 and MCP-1 in cutaneous tissues, but barrier film spray had no effect Furthermore, both topical treatments alleviated the RT-induced response 7 days after irradiation.

Real-time RT-PCR was utilized to quantify the expression of cytokines induced by radiation and changes in expression after topical treatment. Low expression levels were noted in unirradiated control mice and there were no significant changes in expression after topical treatment (unirradiated F-mice and S-mice). Irradiation (15 Gy) induced a significant increase in the mRNA levels of the cytokines detected in the experimental groups compared with the unirradiated group after 24 h. Treatment with steroid cream significantly attenuated the increase of pro-inflammatory cytokines in cutaneous tissues; barrier film spray had no effect (Figure 2a). Western blotting of irradiated skin specimens 24 h after irradiation was used to examine the expression of COX-2, IL-6 and TGF-β1, which are important mediators of radiation-induced inflammation. Topical steroid cream decreased radiation-induced increases in COX-2, IL-6 and TGF-B1expression but barrier film spray had no effect (Figure 2b). Furthermore, analysis of specimens seven days after irradiation demonstrated that both topical treatments alleviated the RT-induced inflammatory response, and that steroid cream had a greater effect than barrier film spray (Figure 3a &3b).

### Effects of topical treatment on the radiation-induced expression of inflammatory response by immunochemical staining

**Figure 4:**
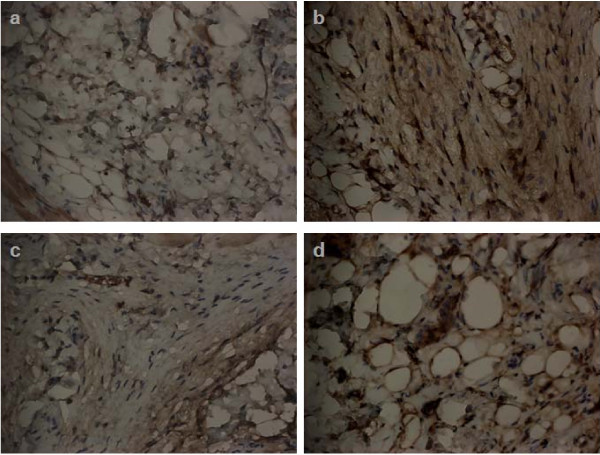
**Effects of topical treatments including steroid and barrier film spray on the radiation- induced injury by immunochemical analysis**. Immunohistochemical analyses using an antibody against TGF-β (Magnification ×400) were performed on murine cutansous tissues 20 days after irradiation. Three mice from each group were examined. Representative slides are shown for (a) unirradiated control mice, (b) irradiated mice at 20 days after 15 Gy irradiation, (c) steroid cream-treated mice at 20 days after 15 Gy irradiation, (d) Barrier film spray-treated mice at 20 days after 15 Gy irradiation. Increased TGF-β immunoreactivity was detected after irradiation. Topical treatment attenuated the inflammatory response, especially in steroid cream treated mice. Duplicate experiments were performed for the analysis.

TGF-β1 has been reported as an important predictive biological marker for RT-induced inflammation and fibrosis [16]. Therefore, we examined TGF-β1 activity by immunochemical analysis under various conditions. As shown in Figure 4, very low levels of TGF-β1 were observed in unirradiated murine skin tissues for each group. Twenty days after exposure to 15 Gy, a pronounced increase in TGF-β1 immunoreactivity was observed in these tissues. A combination of irradiation with topical treatment resulted in a decrease in TGF-β1 immunoreactivity compared with radiation treatment alone, and the effect was more apparent in those treated with steroid cream than in those treated with barrier film spray.

## Discussion

Postoperative radiotherapy has become an integral part of the complex treatment of breast cancer. The risk of acute and late RT-induced sequelae increases with radiation exposure of the organ at risk. The development of appropriate methods for preventing and treating established radiation-induced skin toxicity would be helpful for approximately 25% of breast cancer patients who develop moist desquamation or ulceration of the irradiated chest wall/breast skin [9,10]. At present, there are still no established methods for decreasing radiation dermatitis. In the present study, we evaluated the effects of dosimetry and prophylactic skin care on radiation-induced skin toxicity.

In recent years, 3D-CRT, IMRT and tomotherapy have become promising treatments to improve conformality and dose homogeneity for adjuvant RT treatment of breast cancer. However, there have been few studies concerning the correlation between dosimetric parameters and the incidence of radiation-induced skin toxicity. In the present study, we demonstrated that a larger volume receiving >53.9 Gy within PTV (PTV-V_107%_) and > 55.4 Gy within treated volume (TV-V_110%_) were significant predictors of RT- induced skin toxicity. The similar percentages of patients with higher PTV-V_107% _and TV-V_110% _may explain why the mode of surgery did not significantly affect the incidence of radiation dermatitis.

In addition to dose inhomogeneity, we found that prophylactic skin care (steroid cream and barrier film spray) provided significant protection from radiation-induced dermatitis. There are still no well established prophylactic treatments to prevent radiation skin toxicity despite some agents are reported to reduce radiation induced dermatitis, including topical vitamin C, creams containing urea and steroid [11-13,17-19]. Topical corticosteroid therapy was reported to significantly reduce acute radiation dermatitis by 2 randomized trials, but Potera [18] demonstrated that topical hydrocortisone had no discernible benefit in preventing dermatitis. In the present study, all patients received chest wall/breast irradiation with the same prescribed dose. The results of our study demonstrate that prophylactic skin care decreases radiation-induced dermatitis, especially in patients with larger TV-V_110%_. For patients in who with TV-V_110% _was less than 5.13%, prophylactic skin care provided no significant benefit. Accordingly, we suggested that prophylactic skin care may be helpful preventing higher dose radiation-induced dermatitis in clinics.

Clinically, cutaneous inflammation after irradiation of normal tissue can lead to both temporary and persistent complications. In mice, early radiation dermatitis usually peaks at 20 days [20]. Several studies have implicated cytokine-mediated inflammation in radiation-induced toxicity [20-22]. RT-induced production of pro-inflammatory cytokines including IL-1β, TNF-α, TGF-β1 and IL-6 have been shown to contribute significantly to the complications associated with radiotherapy [23-26]. Early overproduction of both pro-inflammatory cytokines and pro-fibrogenic TGF-β1 during radiotherapy in animal studies suggests a role in the development of acute and late radiation toxicities [16]. Furthermore, TGF-β1 is the master switch cytokine, which once activated after radiation treatment promotes a chain of cellular events that result in radiation-induced fibrosis [27]. In humans, some clinical reports have shown changes in the plasma concentrations of TGF-β1 and IL-6 proteins during radiotherapy, suggesting that these variations could identify patients at risk of radiation toxicity [28,29]. Several studies have also reported COX-2 to be an important gene mediating the subsequent inflammation [30-32]. Such data indicate that the RT-induced response *in vivo *is associated with increased TGF-β1 and COX-2 expression, and inflammatory cytokines. Therefore, we assessed the potential of treatment with topical steroid cream to mitigate skin toxicity caused by irradiation in animal studies. Topical steroid cream decreased the RT-induced inflammatory response, causing a reduction in levels of the pro-inflammatory cytokines IL-1α/β, TNF-α, TGF-ß1, IL-6 and MCP-1. Prophylactic barrier film spray had no effect on the production of early inflammatory cytokines after radiation exposure in mice. However, analysis of inflammatory cytokine RNA, TGF-β1 and COX-2 protein expression one week after irradiation, and TGF-β1immunochemical staining 20 days after irradiation, demonstrated that both topical steroid and barrier film spray did have an impact on the mitigation of radiation dermatitis. Based on our clinical data and experiments *in vivo*, we suggest that prophylactic topical steroid treatment inhibits RT-induced inflammation, leading to decreased radiation dermatitis. The mechanism responsible to barrier film spray- induced decrease in radiation dermatitis might be to decrease skin trauma and skin irritation. However, the effects of barrier film spay on irradiated skin and the underlying mechanisms still need further investigation.

## Conclusions

Our results suggest that dose inhomogeneity as measured by PTV-V_107% _and TV-V_110% _have a significant impact on radiation-induced dermatitis. By multivariate analysis, TV-V_110% _> 5.13% is an important predictor of the incidence of moist desquamation. In addition, prophylactic topical treatment for irradiated skin could significantly improve the tolerance of skin to adjuvant radiotherapy, especially for patients with higher TV-V_110%_. However, due to the limitation of retrospective study, a larger study and a randomized trial are needed to confirm these findings.

## Competing interests

There is no conflict of interest that could be perceived as prejudicing the impartiality of the research reported

## Authors' contributions

MFC conceived of the study, performed the study, drafted the manuscript and participated in coordination. WCC participated in coordination and assisted in editing of manuscript. CHL helped in the collection and analysis of clinical data. CHH and KCL performed statistical analysis and participated in its design YHC helped in the collection of clinical data. All authors read and approved the final manuscript.

## Pre-publication history

The pre-publication history for this paper can be accessed here:

http://www.biomedcentral.com/1471-2407/10/508/prepub
